# Role of disturbed fatty acids metabolism in the pathophysiology of diabetic erectile dysfunction

**DOI:** 10.1186/s12944-017-0637-9

**Published:** 2017-12-12

**Authors:** Mohamed Raâfet Ben Khedher, Houda Bouhajja, Samia Haj Ahmed, Mohamed Abid, Kamel Jamoussi, Mohamed Hammami

**Affiliations:** 1 Laboratory of Nutrition, Functional Food and Vascular Health, Department of Biochemistry, Faculty of Medicine, University of Monastir, Avenue Avicenne, 5019 Monastir, Tunisia; 2 Unit of Obesity and Metabolic Syndrome, Department of Endocrinology, University Hospital Hedi Chaker of Sfax, Sfax, Tunisia; 3 Biochemistry Laboratory, University Hospital Hedi Chaker of Sfax, Sfax, Tunisia

**Keywords:** Diabetic erectile dysfunction, Fatty acids, Inflammation, Insulin resistance, Type 2 diabetes

## Abstract

**Background:**

Vasculogenic erectile dysfunction (VED) is considered as a common complication among people with type 2 diabetes (T2D). We tested whether changes in fatty acid (FAs) classes measured in erythrocytes are associated with increased risk of diabetic VED along with related risk factors.

**Methods:**

We assessed erythrocyte FAs composition, lipid peroxidation parameters and inflammatory cytokines among 72 T2D men with VED, 78 T2D men without VED and 88 healthy volunteers with similar age. Biochemical, hepatic, lipid and hormonal profiles were measured.

**Results:**

T2D people with VED had significant decrease in the indexes of Δ6-desaturase and elongase activities compared to the other studied groups. The same group of participants displayed lower erythrocytes levels of dihomo-γ-linolenic acid (C20:3n-6) (*P* < .001), precursor of the messenger molecule PGE1 mainly involved in promoting erection. Moreover, absolute SFAs concentration and HOMA IR levels were higher in T2D people with VED when compared to controls and associated with impaired NO concentration (1.43 vs 3.30 ng/L, *P* < .001). Our results showed that IL-6 and TNF-α were significantly increased and positively correlated with MDA levels only in T2D people with VED (*r* = 0.884, *P* = .016 and *r* = 0.753, *P* = .035; respectively) suggesting a decrease in the relative availability of vasodilator mediators and an activation of vasoconstrictors release.

**Conclusion:**

Our findings show that the deranged FAs metabolism represents a potential marker of VED in progress, or at least an indicator of increased risk within men with T2D.

## Background

Biomarkers of fatty acid (FAs) intake have been widely used in epidemiologic studies to predict risk of diseases [1]. The reliability and validity of diet records and food frequency questionnaire remain to be proved. Biomarkers of dietary FAs are objective, do not rely on the accuracy of memories and adequately reflect temporal changes in food composition, which could not be readily accommodated by classic methods of dietary assessment [1].

Type 2 diabetes (T2D) is determined chiefly by genes and lifestyle, yet dietary composition may affect both its development and complications [2]. FAs intake may increase diabetes complications risk since it influence glucose metabolism by altering cell enzyme activity, membrane function, gene expression, and insulin signaling [3]. Erectile dysfunction (ED) is one of the major complications of T2D [4]. The development of ED in men with diabetes is a multifactorial and complex process that appears to be affected by neurogenic and vascular causes, changes in nitric-oxide (NO) system and oxidative processes [5].

Dysregulation of FAs metabolism is a key event responsible of insulin resistance (IR) and T2D [6]. In people with T2D, IR inhibits the activation and the expression of endothelial nitric oxide synthase (eNOS) and results in impaired NO bioactivity [7]. NO is released in vascular endothelium by activation of eNOS. Endothelial-derived NO diffuses into adjacent vascular smooth muscle cells (VSMCs) where it reduces intracellular calcium (Ca^2+^) bioavailability secondary to NO-mediated soluble guanylate cyclase (sGC) activation and cyclic guanosine 3′,5′-monophosphate (cGMP) formation and leads to vasorelaxation by increased blood flow [8]. Erection can also be mediated by prostaglandin E1 (PGE1), a messenger molecule released by the cleavage of long chain n-6 dihomo-γ-linolenic acid (DGLA, C20:3) [9]. PGE1 activate the secretion of cyclic adenosine 3′,5′-monophosphate (cAMP) in VSMCs which regulates the activity of intracellular contractile proteins and promote the relaxation of penile VSMCs [9].

Oxidative stress plays a pivotal role in the development of diabetes complications. The involvement of lipid peroxidation in increased diabetes complications risk is clearly demonstrated; however no particular interest was given to its involvement in promoting ED. Free radical formation in diabetes by increased lipid peroxidation leads to damage of cellular machinery, enzymes and increased IR [10]. An excess of free radicals inactivates NO [11]. Yadav and Ramana (2013) showed that some lipid peroxidation products generated following oxidative processes modulate several pro-inflammatory cytokines gene expression [12]. Cytokines interact with VSMCs to activate potent vasoconstrictors release (protein kinase C, rho-kinase) and to inhibit the synthesis of vasodilator mediators (NO, bradykinin) [13].

Recently, it was proposed that eicosapentaenoic acid (EPA, C20:5 n − 3), docosahexaenoic acid (DHA, C22:6 n-3) and their lipid mediator metabolites (resolvins and protectins) possess potent anti-inflammatory and protective effects in diverse diseases including diabetes and its related complications [14].

These different data suggest that substantial dysfunction of FAs metabolism in T2D may play an active role in the pathogenesis of ED. There are no published studies, to the best of our knowledge, which have examined the composition of FAs in red blood cells (RBCs) membranes and ED risk in diabetic patients. The present paper investigated whether changes in FAs metabolism are associated with VED susceptibility and related risk factors in people with T2D.

## Methods

### Vasculogenic erectile dysfunction assessment

The present work is a cross-sectional study of factors associated with a high risk for VED in men with T2D. All subjects diagnosed with T2D as per World Health Organization criteria completed a 5-item International Index of Erectile Function (IIEF-5) questionnaire [15]. IIEF-5 is a diagnostic tool used for the screening of erection problems and for assessing the severity of ED. Patients with a cutoff score of 21 underwent a penile color duplex Doppler ultrasonography test to establish the diagnosis of the type of vascular insufficiency. The assessment of penile vasculature was computed by measuring peak systolic velocity (PSV) in the deep penile arteries and end-diastolic velocity (EDV) after intracavernosal injection with 20 μg prostaglandin E1. A PSV < 25 cm/s or 25–30 cm/s is considered to prove arterial insufficiency, a PSV < 25 cm/s + EDV > 5 cm/s indicate mixed vascular disorder and a PSV > 30 cm/s + EDV ≤ 5 cm/s represent non-vascular disorder [16].

### Study subjects

Patients with any known thyroid dysfunction, hormonal disorder, malformations of the genitalia, Peyronie’s disease, other sexual dysfunction including premature ejaculation or decreased libido, a history of pelvic surgery, kidney and liver failure, and any use of medication known to cause ED, such as antiandrogens, antihypertensive, antiarrhythmics, psychotropic drugs and antidepressants were excluded from the study [17]. According to the IIEF-5 score, the penile color ultrasound parameters and exclusion criteria, 72 men with T2D were diagnosed with VED.

A total of 78 T2D patients without VED were included in the study with respect to the excluding criteria. A number of 88 age-matched healthy volunteers without sexual dysfunction and who did not have diabetes or a history of, psychological, medical or neurological diseases, including ED, participated in this study.

### Ethics statement

All participants gave consent to this study that was approved by the local Medical Ethics Committee of the Hedi Chaker Hospital of Sfax.

### Blood sampling and laboratory methods

Blood samples were collected following overnight fasting. RBCs and plasma were separated by centrifugation. Biochemical, lipid and hepatic profiles were determined by automated analysis using the PLC ADVIA 1800 _CHEMESTRY SYSTEM_. Serum hormones were assessed using assay kits from Abbott laboratories (Chicago, USA) on the Architect Abbott ci4100 analyzer. HbA1c was measured by the PLC BioRAD D-10 (REF: 220-0101, D-10™ Hemoglobin A1C Reorder Pack). Cytokines (BMS213/2 and BMS223/4, eBioscience), insulin (REF:IS130D, Calbiotech) and NO (Cat.no.23479; Sigma) were quantified photometrically using immunoenzymetric assay kits. MDA and conjugated dienes were determined as described previously [18, 19].

### Fatty acids extraction and gas chromatography (GC) analysis

Lipid extraction for quantitative analysis of FAs classes was performed as reported previously [20]. Total lipids fractions were converted into methyl esters using 14% methanolboron trifluoride. The FAs methyl esters were separated, identified and determined by gas chromatography (GC) using a Hewlett Packard 5890 II GC equipped with Flame Ionization Detector and HP-INNOWax capillary column (5% phenyl/95% dimethylpolysiloxane: 30 m × 0.25 mm, *i.d*., film thickness 0.25 μm). The concentration of each individual FAs was expressed as a relative percentage of total area under the peaks according to Zarrouk et al. [21].

### Statistical analysis

The Statistical Package for the Social Sciences (SPPS 18.0 for Windows) was used to perform all statistical analysis. Student’s unpaired *t*-test was used to analyze clinical characteristics. Pearson’s chi-squared test with Fisher’s exact probability was used to compare categorical variables of subject’s baseline data. Mann–Whitney U-test was used in cases that did not have a normal distribution. The difference was considered significant at *P* < .05.

## Results

### Baseline and specific cohort characteristics

The main clinical features, biochemical, inflammatory and hormonal parameters of the studied groups are summarized (Tables 1 and 2). As a known risk factor for ED, age was matched to patients and healthy volunteers groups. A statistically significant increase was observed in both T2D patient groups (Group A: T2D with VED and group B: T2D without VED) compared to the controls (group C) on the rate of fasting glucose, HbA1c, insulin, HOMA IR, total cholesterol and LDL-cholesterol plasmatic levels. However, a significant decrease in both patient groups was observed on ALT levels, when compared to controls. AST concentration was statistically reduced only in patients of group B whereas triglycerides levels were higher in the same group of patients compared to the control group.

**Table 1:** Clinical and biochemical characteristics of the study participants

Characteristics	Patients with VED (*n* = 72)	Patients w/o VED (*n* = 78)	Controls (*n* = 88)
Clinical profile			
Age (years)	57.7 ± 5.1	58.8 ± 4.8	55.9 ± 7.0
BMI (kg/m^2^)	26.75 ± 4.8	27.43 ± 3.2	25.15 ± 3.8
Duration of T2D (years)	10.95 ± 7.1	8.25 ± 5.4	__
Duration of ED (years)	3.30 ± 2.9	__	__
Biochemical profile			
Fasting Glucose (mmoL/L)	9.87 ± 3.7**	8.36 ± 2.1**	5.27 ± 0.9
HbA1c (mmoL/mol)	95**	77**	38
Insulin (μIU/mL)	5.62 ± 3.6*	4.93 ± 2.7*	3.56 ± 2.4
HOMA IR	2.39 ± 1.7**	1.83 ± 1.1**	0.83 ± 0.4
NO (ng/L)	1.43 ± 1.18***	3.09 ± 1.85	3.30 ± 2.92
Serum lipid profile (mmoL/L)			
Total cholesterol	4.54 ± 0.8*	4.43 ± 0.8*	4.06 ± 0.9
Triglycerides	1.60 ± 0.9	1.77 ± 0.7*	1.37 ± 0.5
LDLc	2.70 ± 0.94*	2.68 ± 0.9*	2.27 ± 1,02
HDLc	1.03 ± 0.3	0.94 ± 0.4	1.1 ± 0.4
Serum Hepatic profile (IU/L)			
AST	23.19 ± 7.5	21.5 ± 8.4*	24.28 ± 8.9
ALT	20.26 ± 11.6*	21.36 ± 10.4*	25.01 ± 12.3

Values are given as mean ± standard error

*BMI* Body mass index, *HbA1c* glycated hemoglobin, *HOMA IR* homeostatic index of insulin resistance, *LDLc* low density lipoprotein-cholesterol, *HDLc* High density lipoprotein-cholesterol, *AST* aspartate aminotransferase, *ALT* alanine aminotransférase, *w/o* without

*P < 0.05 and **P < 0.001 compared to controls

**Table 2:** Specific clinical characteristics of studied subjects

Characteristics	Patients with VED (*n* = 72)	Patients w/o ED (*n* = 78)	Controls (*n* = 88)
Inflammatory Cytokines			
IL-6 (pg/mL)	0.86 ± 0.5**	0.51 ± 0.2	0.44 ± 0.3
TNF-α (pg/mL)	3.3 ± 2.9**	2.9 ± 1.9**	1.43 ± 1.2
Lipid peroxidation			
MDA (μM)	23.9 ± 4.5**	18.7 ± 5.3	17.11 ± 5.9
Conjugated diene (μM)	139.16 ± 68.4	141.76 ± 34.5	122.4 ± 55.9
Serum Hormones			
FSH (mIU/mL)	7.22 ± 4.9	6.83 ± 2.6	6.32 ± 2.4
LH (mIU/mL)	5.67 ± 3.4	6.14 ± 3.6	6.07 ± 2.1
PRL (ng/mL)	12.16 ± 5.1	12.83 ± 4.4	11.19 ± 2.9
Testosterone (ng/mL)	5.35 ± 1.5	6.12 ± 2.8	5.89 ± 2.1
FT4 (pmoL/L)	13.92 ± 1.5	12.53 ± 2.6	13.64 ± 2.3
hsTSH (μIU/mL)	1.95 ± 0.9	1.76 ± 0.7	1.83 ± 0.9

Values are given as mean ± standard error

*NO* nitric oxide, *TNF-α* tumor necrosis factor alpha, *IL-6* interleukine 6, *MDA* malondialdehyde, *FSH* follicle-stimulating hormone, *LH* luteinizing hormone, *PRL* prolactin, *FT4* Free thyroxine 4, *hsTSH* high sensitive thyroid-stimulating hormone, *w/o* without

*P < 0.05 and **P < 0.001 compared to controls

Mean concentrations of MDA, IL-6 and TNF-α were significantly higher in group A than the control group (Table 2). Our results showed that IL-6 and TNF-α were positively correlated with MDA levels only in T2D patients of group A (*r* = 0.884, *P* = .016 and *r* = 0.753, *P* = .035; respectively) (Table 3). The group A also displayed lower plasmatic levels of NO compared to the other groups. No differences in conjugated diene concentrations and all tested serum hormones (FSH, LH, PRL, testosterone, FT4 and hsTSH) were recorded between the three studied groups.

**Table 3:** Univariate correlation of malondialdehyde with inflammatory cytokines levels among the study population

Inflammatory Cytokines	Studied Subjects	Correlation with MDA	
		r	*p*
IL-6	Patients with VED	0.884	0.016*
	Patients w/o VED	−0.184	0.497
	Controls	−0.121	0.312
TNF-α	Patients with VED	0.753	0.035*
	Patients w/o VED	0.497	0.078
	Controls	−0.116	0.333

*IL-6* interleukine 6, *TNF-α* tumor necrosis factor alpha, *MDA* malondialdehyde, *r* correlation coefficients, *w/o* without

*P < 0.05

### Evaluation of fatty acid profile in red blood cells

The FAs profile of RBC membranes and the indexes of the microsomal enzymes systems activities are given in Table 4. Analysis of FAs composition in people with T2D of group A and B showed a significant increase in the levels of very long chain fatty acids (VLCFA) such as behenic acid (C22:0), lignoceric acid (C24:0) and cerotic acid (C26:0) compared to healthy subjects. Besides, T2D patients of group A presented higher total saturated fatty acids (SFAs) content than patients of group B and the controls mainly due to the increase of VLCFA, myristic and palmitic acids. Meanwhile, total monounsaturated FAs (MUFAs) concentration was lower in T2D patients of group A and B than in controls with most particularly a decrease in myristoleic acid (C14:1 n-5), oleic acid (C18:1 n-9) and eicosenoic acid (C20:1 n-9).

**Table 4:** Erythrocytes fatty acid profiles of the study population determined by gas chromatography

Fatty Acids (nomenclature)	Name	Patients with VED (%) (*n* = 72)	Patients w/o VED (%) (*n* = 78)	Controls (%) (*n* = 88)
C12: 0	Lauric acid	0.21 ± 0.12	0.19 ± 0.11	0.21 ± 0.13
C13: 0	Tridecylic acid	0.79 ± 0.53**	0.47 ± 0.45	0.53 ± 0.35
C14: 0	Myristic acid	4.12 ± 2.50***	3.03 ± 2.09*	2.57 ± 2.30
C15: 0	Pentadecylic acid	1.07 ± 0.69***	0.75 ± 0.45*	0.63 ± 0.51
C16: 0	Palmitic acid	28.82 ± 5.75**	29. 25 ± 6.66**	25.87 ± 4.16
C18: 0	Stearic acid	11.93 ± 5.82***	13.29 ± 5.18*	14.88 ± 5.15
C20: 0	Arachidic acid	0.24 ± 0.32	0.23 ± 0.13*	0.26 ± 0.31
C22: 0	Behenic acid	0.43 ± 0.41***	0.35 ± 0.16**	0.27 ± 0.21
C23: 0	Tricosylic acid	0.59 ± 0.42	0.44 ± 0.28	0.60 ± 0.79
C24: 0	Lignoceric acid	2.06 ± 0.83**	1.75 ± 1.14	1.42 ± 1.19
C26: 0	Cerotic acid	5.47 ± 2.28***	3.21 ± 3.05**	3.72 ± 2.81
Σ SFA		55.70 ± 9.4**	52.84 ± 7.1	51,01 ± 8.7
C14:1 cis-9 (n-5)	Myristoleic acid	1.33 ± 0.71**	0.98 ± 0.44***	1.60 ± 0.56
C16:1 cis-9 (n-7)	Palmitoleic acid	0.56 ± 0.51	0.60 ± 0.54	0.57 ± 0.47
C18:1 cis-9 (n-9)	Oleic acid	10.96 ± 4.18***	13.14 ± 3.31*	14.53 ± 3.65
C18:1 cis 11(n-7)	Vaccenic acid	1.31 ± 0.30	1.39 ± 0.38	1.47 ± 0.49
C20:1 cis11 (n-9)	Eicosenoic acid	0.16 ± 0.13***	0.22 ± 0.18*	0.26 ± 0.17
C22:1 cis13(n-9)	Erucic acid	0.45 ± 0.21	0.50 ± 0.21	0.54 ± 0.87
C24:1 (n-9)	Nervonic acid	1.70 ± 0.59***	1.61 ± 0.97***	3.00 ± 1.31
Σ MUFA		16.50 ± 4.12***	18.43 ± 3.65***	21.00 ± 3.88
Omega 3 family (n-3)				
C18:3 cis9,12,15	α-linolenic acid	0.66 ± 0.34***	0.63 ± 0.27***	0.51 ± 0.28
C20:3 cis11,14,17	Eicosatrienoic acid	1.43 ± 0.48	1.80 ± 0.83*	1.61 ± 0.95
C20:5 EPA	Eicosapentaenoic acid	0.52 ± 0.26***	1.26 ± 1.02	1.54 ± 1.92
C22:6 DHA	Docosahexaenoic acid	2.00 ± 1.39***	2.27 ± 1,21***	3.13 ± 1.20
Omega 6 family (n-6)				
C18:2 cis9,12	Linoleic acid	12.05 ± 4.46***	10.40 ± 3.20**	9.40 ± 5.21
C18:3 cis6,9,12	γ-linolenic acid	0.16 ± 0.17*	0.19 ± 0.14	0.17 ± 0.11
C20:3 cis8,11,14	Dihomo-γlinolenic acid	0.18 ± 0.09***	0.28 ± 0.16	0.38 ± 0.27
C20:4	Arachidonic acid	10.75 ± 3.09*	11.43 ± 2.38***	9.25 ± 3.36
Σ PUFA		27.79 ± 5.06	28.42 ± 4.11	26.03 ± 5.85
Σ UFA		44.30 ± 5.75**	47.16 ± 6.03	47.03 ± 6.75
Desaturation index				
C20:4/C20:3 (Δ5D)		77.03 ± 51.26***	54.18 ± 40.75*	47.99 ± 43.92
C18:3/C18:2n-6 (Δ6D)		0.015 ± 0.016**	0.020 ± 0.015	0.023 ± 0.021
C16:1/C16:0 (Δ9D-16)		0.02 ± 0.02	0.02 ± 0.02	0.02 ± 0.01
C18:1/C18:0 (Δ9D-18)		1.18 ± 0.87	1.17 ± 0.66	1.17 ± 0.71
C24:1/C18:1		0.18 ± 0.11*	0.12 ± 0.08***	0.22 ± 0.12
C18:0/C16:0		0.41 ± 0.21***	0.48 ± 0.21**	0.57 ± 0.17

Data obtained by GC are expressed as relative values (%): mean % of total FAs ± SD; **Σ** SFA, sum of saturated fatty acids; **Σ** MUFA, sum of monounsaturated fatty acids; **Σ** PUFA, sum of polyunsaturated fatty acids; **Σ** UFA, sum of unsaturated fatty acids; C20:4/C20:3, Δ5 desaturation index; C18:3/C18:2, Δ6 desaturation index; C16:1/C16:0 and C18:1/C18:0, Δ9 desaturation index; C24:1/C18:1 and C18:0/C16:0, elongation index; w/o, without

*P < 0.05; **P < 0.01 and ***P < 0.001 compared to controls. The Mann-Whitney test with Bonferroni correction was used

The data on the composition of FAs subtypes of PUFAs showed significant changes, however absolute PUFAs concentration was similar in all groups. The differences reported in this lipid fraction were primarily due to the increases of ώ3 α-linolenic acid (ALA, C18:3 n-3) and of ώ6 linoleic acid (LA, C18:2 n-6) in patients of group A compared to controls. However, a significant decrease was recorded in the same group of patients regarding erythrocytes concentration of dihomo-γ-linolenic acid (DGLA, C20:3 n-6), EPA (C20:5 n-3) and DHA (C22:6 n-3).

The desaturation indexes of enzymes activities were assessed as described previously [20]. The elongase activity was statistically lower in patients of group A and B compared to the control group. The index of Δ6-desaturase activity was substantially reduced only in patients with VED compared to the control subjects. A significant increase was noted for Δ5-desaturase activity in both T2D patients groups in comparison to healthy volunteers. No variations were recorded for the Δ9-desaturase activity between the studied groups.

### Additional study variables

The patients were divided into four groups according to the severity of ED: 7 (9.8%) had light ED, 29 (40.4%) had mild ED, 24 (33.4%) had moderate ED and 12 (16.4%) had severe ED. The distribution of FAs classes according to ED severity was assessed using a multivariate analysis. No significant associations were recorded between the IIEF-5 score and FAs profiles. Similarly, significantly different onset of age of VED was not found between the different FAs classes (data not shown).

## Discussion

The physiopathology of diabetic ED is multifactorial and a number of associated risk factors are proposed [5], yet new molecular and cellular approaches are suspected to promote this complication. The present paper aims to investigate the effect of disturbed FAs metabolism and obvious risk factors on the susceptibility to VED in people with T2D.

**Fig. 1:**
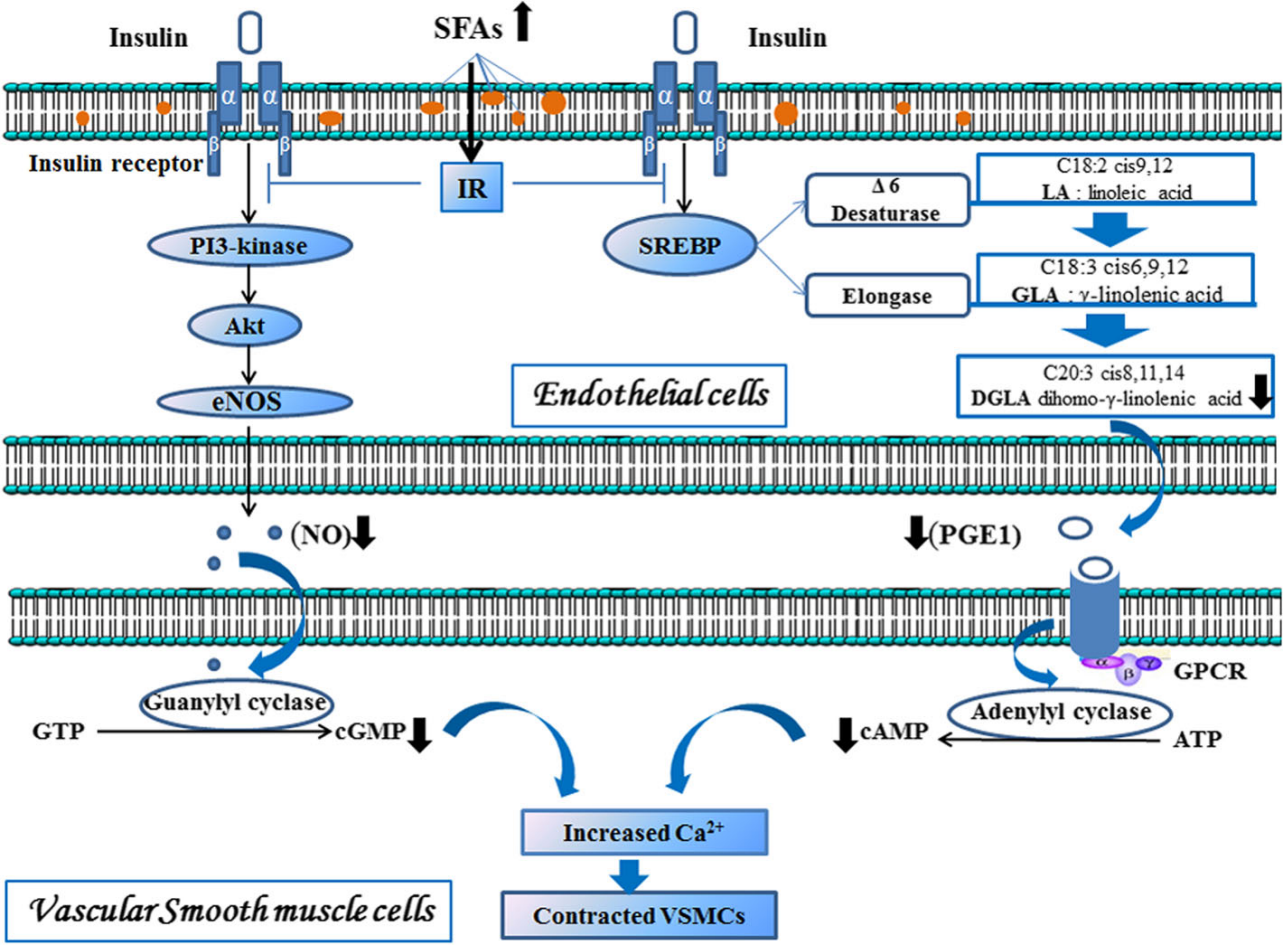
Hypothetical interaction between disturbed FAs metabolism and IR leading to impaired penile erection. SFAs, Saturated Fatty Acids; IR, Insulin Resistance; PI3-kinase, Phosphatidylinositol 3-Kinase; SREBP, Sterol Regulatory Element Binding Protein; Akt, Protein kinase B; eNOS, Endothelial Nitric Oxide Synthase; PGE1, Prostaglandin E1; NO, Nitric Oxide; GPCR, G-Protein-Coupled Receptor; ATP, Adenosine Triphosphate; cAMP, cyclic Adenosine Monophosphate; GTP, Guanosine Triphosphate; cGMP, cyclic Guanosine Monophosphate; Ca^2+^, Calcium; VSMCs, Vascular Smooth Muscle Cells; (), Increase; (), Decrease

Insulin has multiple physiological effects on the vascular tissues such as vasodilation, mainly due to its ability to increases eNOS expression and NO production via the activation of PI3K/Akt pathways [8]. However, IR prevents this increase [7]. The hypothesis that IR is a result of accumulation of intracellular lipid metabolites (e.g., FAs, diacylglycerol, fatty acyl CoAs) in endothelial cells, hepatocytes and skeletal muscle is supported by observations in patients and mouse models of lipodystrophy [22]. In fact, high SFAs amount affects the integrity, the fluidity and the structure of cell membranes leading to a decrease in the number of insulin receptors and an increase of IR severity [23] (Fig. 1).

In the present study, patients with VED displayed higher SFAs content associated with increased HOMA-IR levels and lower concentration of NO than the other studied groups. Interestingly, this decline in NO levels was detected in T2D patients with VED (A) but not in those without VED (B), although both of the groups had IR. The severity of IR may play a crucial role in impaired NO bioactivity. As shown in our study, patients of group A developed diabetes during a longer period (Table 1) and had a greater amount in absolute SFAs concentration (Table 4) than patients of group B. This finding suggests that the length of the disease process, the excess of SFAs content and IR are the main culprits that affect the NO-cGMP pathway leading to changes in vascular tone and VSMCs function.

The synthesis of PUFAs requires several reactions of elongation catalyzed by the microsomal enzymes systems, essentially elongase, delta-5 (Δ5D) and delta-6 desaturases (Δ6D) [24]. In fact, Δ6D catalyzes the conversion of LA (C18:2 n-6) into GLA (18:3 n-6), whereas elongase converts GLA (18:3 n-6) into DGLA (20:3 n-6). The outputs of DGLA and precisely its metabolite PGE1 activates soluble adenylyl cyclase (sAC), which increase cAMP concentration. Acting as a second messenger molecule, cAMP decrease intracellular calcium concentration and participate in VSMCs relaxation [10]. Our study reveals that T2D patients with VED have significantly lower indexes of elongase and Δ6-desaturase activities than the control group as well as a significant decrease in DGLA levels. It thereby affirms that the stimulation of erection through the PGE1-cAMP pathway is down regulated. The suggested mechanism underlying the observed decrease in the index of elongase and Δ6D activities is IR. In fact, insulin modulates the activities of some enzymes of the microsomal systems [25]. Insulin effects occur via the transcriptional activation of lipogenic transcription factors such as sterol regulatory element binding protein (SREBP), which increase the transcription of genes encoding enzymes of PUFAs synthesis such as Δ6-desaturase, acetyl–coenzyme A (CoA) carboxylase, fatty acid synthase, and the elongase [25, 26] (Fig. 1). Accordingly, IR plays an essential role in inhibiting the activity of these enzymes affecting afterwards DGLA synthesis and PGE1-mediated sAC activation.Diet constitutes a crucial aspect of the overall management of diabetes, which may prevent or ameliorate diabetes-related complications. In fact, recent studies suggested that PUFAs intake lowers the risk of vascular complications associated with diabetes such as retinopathy [27] and nephropathy [28]. A multicenter clinical trial revealed a significant improvement of 13 diabetic neuropathy parameters in T2D patients supplemented with gamma-linolenic acid for one year [29]. Moreover, it was demonstrated that an excess of SFAs content is positively correlated with IR and T2D risk, whereas long chain n-3 FAs such as EPA and DHA are protective [18, 30]. These two PUFAs can be synthesized from ALA (18:3 n-3). Although RBCs levels of ALA are higher in patients of group A, a decrease in EPA and DHA concentrations is recorded. Research suggests that only a small amount of these two biologically important dietary FAs can be synthesized in the body from ALA process [31] and our study showed that the efficiency of the enzymatic reactions involved is rather low., Furthermore, Delarue and Guriec (2011) reported that diet rich in MUFAs, improved insulin sensitivity in patients with T2D [30]. MUFAs reduce plasma triglycerides, postprandial lipemia and markers of inflammation [30]. In the present study a significant reduction in erythrocytes levels of several ώ9 MUFAs were registered among T2D patients with VED. The findings herein reported obviously corroborate the fact that imbalanced diet fat intake may be involved in the physiopathology of VED in people with T2D. We believe that it would be interesting to test the potential beneficial effects of DGLA n-6 supplementation with adequate amounts of PUFAs (such as EPA and DHA) on modulating the risk of this disorder.

Nuclear transcription factors involved in the induction of cytokine genes are regulated by lipid mediators and products of lipid peroxidation [32]. MDA is a main ώ6 PUFAs lipid peroxidation product that up-regulates IL-6 gene expression [32]. The stimulation of TNF-α/IL-6 signaling is a key mechanism underlying increased Endothelin-1 (ET-1) production and subsequent vasoconstriction effects [33]. ET-1 is one of the most powerful endogenous vasoconstrictors. Moreover, the levels of eNOS mRNA in human endothelial cells (ECs) are directly decreased by high concentrations of TNF-α [34]. There is evidence that cytokines activation in VSMCs hinders the production and/or activity of vasodilator mediators such as NO, bradykinin, prostacyclin and triggers vasoconstrictive mediators expression such as ET-1, thromboxane and angiotensin II [13]. In the present investigation, MDA and IL-6/TNF-α plasmatic levels are significantly higher and positively correlated in T2D patients with VED. Altogether, our results support a major role of inflammation in the pathogenesis of diabetic VED.

### Strengths and limitations

FAs can be measured in various tissues and blood fractions such as RBCs, serum or plasma. FAs composition in membranes of RBCs may be superior to plasma or serum FAs for reflecting long-term FAs intake because of a slower turnover rate and less sensitivity to recent intake [1]. Hence, RBCs represent the most suitable model for comparing FAs classes with regard to their effect on metabolic disorders.

The limitations of the present study should be regarded. The sample size is adequate for statistical analysis but not large enough for epidemiological considerations. The measurement of desaturases activities in the leukocytes and SHBG (sex hormone binding globulin) level should be considered in future investigations. A dietary survey is recommended to support our findings.

## Conclusions

The conducted study shows that balanced FAs metabolism is required for the regulation of key pathways involved in stimulating erection. Understanding the cellular mechanism(s) generating FAs dysregulation offers the prospect of better targeted and more effective therapeutic interventions for treatment and prevention of VED in men with T2D.
